# Virulence Characterisation of *Salmonella enterica* Isolates of Differing Antimicrobial Resistance Recovered from UK Livestock and Imported Meat Samples

**DOI:** 10.3389/fmicb.2016.00640

**Published:** 2016-05-02

**Authors:** Roderick Card, Kelly Vaughan, Mary Bagnall, John Spiropoulos, William Cooley, Tony Strickland, Rob Davies, Muna F. Anjum

**Affiliations:** ^1^Department of Bacteriology, Animal and Plant Health AgencyWoking, UK; ^2^Department of Pathology, Animal and Plant Health AgencyWoking, UK

**Keywords:** *Salmonella enterica*, virulence, *Galleria mellonella*, antimicrobial resistance, livestock

## Abstract

*Salmonella enterica* is a foodborne zoonotic pathogen of significant public health concern. We have characterized the virulence and antimicrobial resistance gene content of 95 *Salmonella* isolates from 11 serovars by DNA microarray recovered from UK livestock or imported meat. Genes encoding resistance to sulphonamides (*sul1*, *sul2*), tetracycline [*tet*(A), *tet*(B)], streptomycin (*strA*, *strB*), aminoglycoside (*aadA1*, *aadA2*), beta-lactam (*bla*_TEM_), and trimethoprim (*dfrA17*) were common. Virulence gene content differed between serovars; *S.* Typhimurium formed two subclades based on virulence plasmid presence. Thirteen isolates were selected by their virulence profile for pathotyping using the *Galleria mellonella* pathogenesis model. Infection with a chicken invasive *S.* Enteritidis or *S.* Gallinarum isolate, a multidrug resistant *S.* Kentucky, or a *S.* Typhimurium DT104 isolate resulted in high mortality of the larvae; notably presence of the virulence plasmid in *S.* Typhimurium was not associated with increased larvae mortality. Histopathological examination showed that infection caused severe damage to the *Galleria* gut structure. Enumeration of intracellular bacteria in the larvae 24 h post-infection showed increases of up to 7 log above the initial inoculum and transmission electron microscopy (TEM) showed bacterial replication in the haemolymph. TEM also revealed the presence of vacuoles containing bacteria in the haemocytes, similar to *Salmonella* containing vacuoles observed in mammalian macrophages; although there was no evidence from our work of bacterial replication within vacuoles. This work shows that microarrays can be used for rapid virulence genotyping of *S. enterica* and that the *Galleria* animal model replicates some aspects of *Salmonella* infection in mammals. These procedures can be used to help inform on the pathogenicity of isolates that may be antibiotic resistant and have scope to aid the assessment of their potential public and animal health risk.

## Introduction

*Salmonella enterica* is a Gram-negative bacterium of significant public and animal health concern worldwide (Majowicz et al., 2010). The species is highly diverse, consisting of over 2,600 serovars (Grimont and Weill, 2007) which can be divided into typhoidal and non-typhoidal *Salmonella* (NTS) serovars. The typhoidal serovars comprise *Salmonella* serovar Typhi, *Salmonella* serovar Sendai, and *Salmonella* serovar Paratyphi A, B (excluding variant Java), or C that are highly adapted to the human host, in which they cause enteric fever (Gal-Mor et al., 2014). NTS comprise the majority of serovars, some of which are adapted to non-human hosts, e.g., *Salmonella* serovar Gallinarum in poultry and *Salmonella* serovar Dublin in cattle; while others have broad host ranges, e.g., *Salmonella* serovar Typhimurium and *Salmonella* serovar Enteritidis (Uzzau et al., 2000). Many NTS are zoonotic and human infections usually arise via a foodborne route (Majowicz et al., 2010), commonly resulting in a self-limiting gastroenteritis but in approximately 3–10% of cases the infection becomes invasive (Hohmann, 2001) resulting in a serious and potentially fatal condition that often requires antimicrobial treatment (Okeke et al., 2005).

The pathogenic capacity of different NTS serovars is dependent upon the virulence potential of the bacterium and the susceptibility of the host. *S. enterica* harbor a diverse assortment of virulence genes required for adhesion, invasion, intra-cellular survival, and replication. These genes are located on various elements of the genome including the chromosome, plasmids, integrated bacteriophage DNA, *Salmonella* pathogenicity islands (SPIs), and *Salmonella* genomic islands (SGIs). Some of these elements are conserved throughout the species, such as SPI-1 (encoding factors important for cell adhesion) and SPI-2 (encoding factors required for intracellular survival and replication); while other elements are serovar specific, e.g., SPI-7 in *S.* Typhi (Anjum et al., 2005). *Salmonella* virulence plasmids have also been identified in several serovars, including *S.* Typhimurium and *S.* Enteritidis, and, although they can vary in genetic content, all carry the *spv* operon which contributes to pathogenesis by promoting intra-macrophage survival (Gulig et al., 1998; Feng et al., 2012; Haneda et al., 2012). The presence of other plasmids harboring antimicrobial resistance (AMR) genes have been described in many serovars (Hopkins et al., 2007; Anjum et al., 2011). Many of these plasmids are readily transmissible between bacteria via conjugation and their presence limits the choice of antimicrobial treatments suitable for treatment. Therefore tools that can easily determine the resistance profile and inform on the potential for pathogenicity of *Salmonella* isolated from food animals or food products can inform on the possible risk posed to public or animal health.

The aim of this study was to screen 95 *S. enterica* isolates recovered from UK livestock or imported meat, and representing 11 NTS serovars, for the presence of virulence and AMR genes by DNA microarray (Card et al., 2015; Figueiredo et al., 2015) and to investigate the virulence potential of selected isolates in an insect model. Isolates included *S.* Typhimurium from different host animals, poultry-invasive isolates of *S.* Enteritidis and *S.* Gallinarum, and a multidrug resistant *Salmonella* serovar Kentucky of Sequence Type 198 which was highly ciprofloxacin resistant and has been epidemiologically associated with outbreaks in poultry and humans in Europe and Africa (Le Hello et al., 2013). The virulence pathotype of 13 isolates was investigated using the *Galleria mellonella* (greater wax moth) pathogenesis model, which we have described previously (Kirchner et al., 2013; Figueiredo et al., 2015). This model has been used to assess virulence of several bacterial species including *S. enterica* (Bender et al., 2013; Viegas et al., 2013; Figueiredo et al., 2015), *Campylobacter jejuni* (Champion et al., 2010; Senior et al., 2011), *Escherichia coli* (Kirchner et al., 2013), and *Legionella pneumophila* (Harding et al., 2012). A good correspondence with mammalian models has been established for *G. mellonella* and its innate immune response has many similarities to that of vertebrates, including the presence of haemocytes that play a similar role to that of macrophages in vertebrates and circulate in the insect haemolymph (Kavanagh and Reeves, 2004). The haemolymph fills the insect body cavity and functions analogously to blood in mammals. In this study we also used light and electron microscopy to examine the histopathology in the larvae following *Salmonella* infection.

## Materials and Methods

### *Salmonella enterica* Isolates Tested

Ninety-five *S. enterica* isolates were selected from diagnostic or surveillance submissions held in the culture collection at APHA (isolates detailed in Supplementary Table S1). The isolates were obtained from livestock or imported meat sources, comprising cattle (*n* = 26), broiler chicken (*n* = 16), layer chicken (*n* = 18), chicken meat (*n* = 3), swine (*n* = 16), turkey (*n* = 13), duck (*n* = 1), and sheep (*n* = 2). Isolates were selected to include serovars with either a broad host range, a high prevalence in specific hosts or importance to human or animal health and comprised: *S*. Typhimurium (including monophasic variants with the antigenic formula 1,4,[5],12:i:-; *n* = 45), *Salmonella* serovar Derby (*n* = 7), *S.* Paratyphi B var. Java (*n* = 2), *Salmonella* serovar Infantis (*n* = 1), *Salmonella* serovar Mbandaka (*n* = 19), *Salmonella* serovar Montevideo (*n* = 7), *S.* Kentucky (*n* = 1), *S*. Dublin (n = 8), *S*. Enteritidis (*n* = 1), *S*. Gallinarum (*n* = 2), and non-motile *S. enterica* with the antigenic formula 9,12:-, but biochemically distinct from *S*. Gallinarum (*n* = 2). The *S.* Typhimurium strain LT2 was also included in this study as a reference strain that has a published genome sequence (McClelland et al., 2001).

### Isolate culture, DNA Extraction, and Microarray

The isolates were recovered from storage on Dorset egg medium by culture on blood agar for ∼24 h at 37°C. For DNA extraction, the isolates were cultured overnight at 37°C on Luria-Bertani (LB) agar. A 10 μl loop of bacteria was lysed and DNA quantified using a nanodrop apparatus, as described previously (Card et al., 2013). For testing on the microarray the DNA was labeled in a linear multiplex reaction using the AMR and virulence gene primers described previously (Card et al., 2015; Figueiredo et al., 2015). For the microarray hybridization procedures, the HybPlus Kit (Alere Technologies, Jena, Germany) was used according to the manufacturer’s instructions with an adapted protocol described previously (Card et al., 2015). Microarray signals were detected with the ArrayMate device (Alere Technologies) using IconoClust software (Standard version; Alere Technologies). Mean signal intensities of two replicate spots per probe were used for analysis. For the AMR gene probes intensities ≥0.6 were considered present and for the virulence gene probes mean signal intensities ≥0.5 were considered present. The microarray data were converted to a binary format whereby 1 indicated gene presence and 0 indicated gene absence. Strains were clustered based on their virulence determinants using the Jaccard similarity coefficient and UPGMA cluster analysis method with Bionumerics software (Version 6.6, Applied Maths, Sint-Martens-Latem, Belgium) to generate a dendrogram.

### *Galleria mellonella* Pathogenesis Model

To determine an effective infectious dose, strain LT2 and the *S.* Typhimurium field isolate S03659-10 were used. Bacterial strains were cultured for approximately 18 h in LB broth with shaking at 37°C. Cells were harvested to give a final concentration of 10^8^ CFU/ml in 0.1 M phosphate-buffered-saline (PBS) (pH 7.2) and were further diluted in PBS in 10-fold dilution series to 10^4^ CFU/ml. For each strain, ten *G. mellonella* larvae were injected with 10 μl of bacterial suspension, from 10^2^ to 10^6^ CFU, using a previously described micro-injection technique (Champion et al., 2010). Larvae were incubated at 37°C and assessed for survival at 24 h post-infection (hpi). Larvae were assessed as ‘dead’ if they displayed no movement in response to touch. The inoculating dose was verified by plating on LB agar. At 24 hpi *S. enterica* was enumerated in three larvae for each isolate as described previously (Kirchner et al., 2013). A further eleven *S. enterica* strains (see Supplementary Table S1 and Results) were tested in the model using a single dose of 10^2^ CFU per larvae, with subsequent verification of the inoculating dose. Each isolate was tested with 10 larvae in three separate experiments and every experiment included, as controls, uninfected and PBS-injected larvae. Survival data were plotted and standard deviations calculated using GraphPad Prism (version 6.04) (GraphPad Software Inc, San Diego, CA, USA). Differences in survival following infection was assessed for significance (*P* < 0.05) using the Welch-corrected 2-tailed *t*-test.

### Time course, Histopathogy, and Transmission Electron Microscopy Following Infection of *Galleria mellonella*

A time-course study was performed to examine the progress of *Salmonella* infection in *G. mellonella* and to investigate the ensuing histopathology. Four isolates (see Supplementary Table S1 and Results) were selected for this based on results obtained from the microarray and the *G. mellonella* model. The time course ran for 24 h with samples collected at 0, 5, 20, and 24 hpi. At each time point five larvae were taken for haemolymph extraction and three for formalin fixation. Larvae were chilled at 4°C for at least 2 h prior to fixation in formalin for 1 week at room temperature. Each larva was cut longitudinally and both parts were embedded in paraffin. Four micron thick histological sections were stained with haematoxylin and eosin or Gram-stain, as previously described (Senior et al., 2011), and examined under a light microscope (Nikon 80i) and photographed using a Nikon Ds-Ri1 digital camera attached to a PC equipped with Nikon NIS-Br imaging software. For each *Salmonella* isolate tested the extent of degeneration of the gut structure in the larvae was recorded as: no change/normal (scored as zero), moderate (scored as 1), or severe (scored as 2) and plotted as the aggregate score obtained from the three formalin fixed larvae at each time-point.

Haemolymph was extracted from five larvae at each time point, pooled and 500 μl 3% glutaraldehyde added, mixed, and transferred to a 1 ml sterile tube. The sample was centrifuged at 1180 *g* for 3 min and the pellet re-suspended in 200 μl of 3% glutaraldehyde. After fixation for at least 24 h the pellets were washed in 0.1 M PBS, post-fixed in 1% osmium tetroxide, dehydrated through a gradual series of alcohol up to 100% alcohol and placed in propylene oxide prior to embedding in araldite resin. The resin was polymerised at 60°C for 48 h. One-micron sections, stained with toludine blue were prepared for light microscopy examination. Areas observed containing bacteria and cells were selected for ultrastructural examination. Ultrathin sections at 70–90 nm thickness were then prepared onto copper grids using a diamond knife, contrasted with uranyl acetate and lead citrate prior to examination using a Tecnai 12 BioTwin (FEI) transmission electron microscope (TEM).

## Results

### Resistance and Virulence Genotyping by Microarray

Sixty-four of the 95 field isolates were positive by microarray for one or more AMR gene (median number of genes = 4); no AMR genes were present in the remaining isolates including LT2 (Supplementary Table S1). Eight isolates harbored ≥10 AMR genes and these were from *S.* Typhimurium *(n* = 6 pig; *n* = 1 cattle) and *S.* Kentucky (*n* = 1 turkey). In total, 26 different AMR genes were detected and the most common genes included: *sul1* and *sul2* (sulphonamide resistance); *tet*(A) and *tet*(B) (tetracycline resistance); *strA* and *strB* (streptomycin resistance); *aadA1* and *aadA2* (aminoglycoside resistance); *bla*_TEM_ (beta-lactam resistance); and *dfrA17* (trimethoprim resistance). Four of these genes, *strB*, *bla*_TEM_, *sul2*, and *tet*(B), were present together in 25 *S.* Typhimurium isolates (of which 24 were DT193). The five resistance genes *bla*_PSE-1_, *floR*, *aadA2*, *sul1*, and *tet*(G), characteristic of SGI-1 (Briggs and Fratamico, 1999), were detected in one *S.* Typhimurium DT104 isolate (S03099-12) from a pig host. The integrase genes from class 1 and class 2 integrons were detected in 14 and 3 isolates, respectively. Seven isolates found positive for the class 1 integron also harbored the genes *aadA1*, *cmlA1*, *dfrA12*, and *sul3*. The two *S.* Paratyphi B var. Java isolates harbored the class 2 integron, *aadA1*, *dfrA1*, *aac6’-aph2’*, and *sul1*.

**FIGURE 1:**
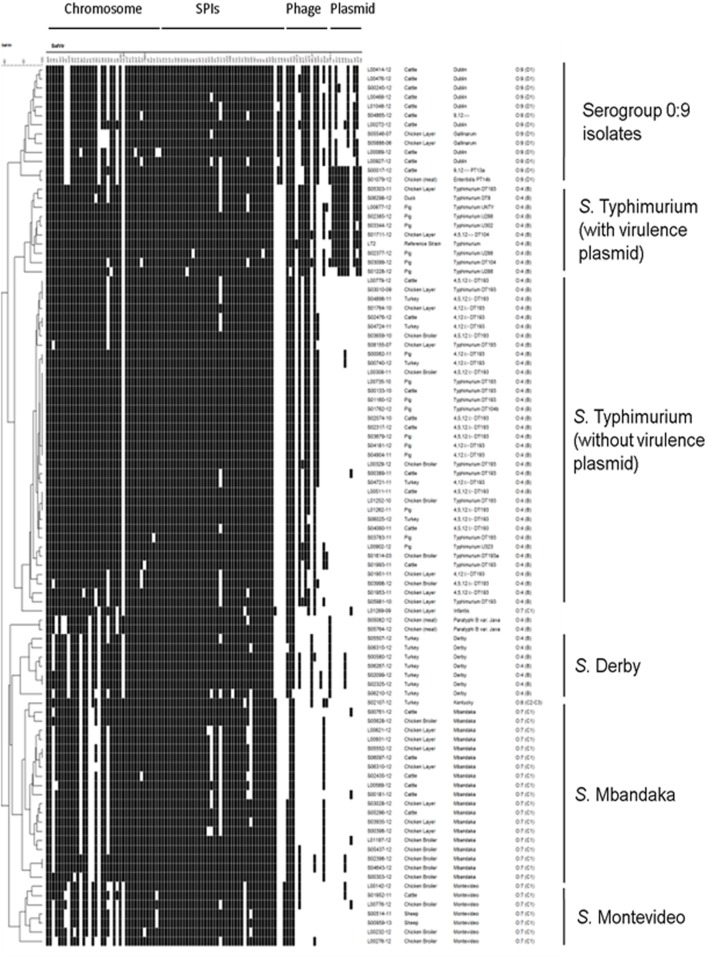
**Virulence determinant microarray data for 95 ***Salmonella enterica*** field isolates and reference strain LT2.** Genes are grouped by genomic location along the top and the hybridization result of each isolate is shown by row. The order of strains represents their relatedness according to UPGMA cluster analysis using Jaccard similarity coefficient performed in BioNumerics 6.6. A white box indicates the absence and a black box indicates the presence of the target sequence in the strain.

A total of 105 *Salmonella* virulence genes were represented on the microarray (Figueiredo et al., 2015) and 40 genes were present in all isolates, two genes were not detected in any isolate while the remaining 63 were variably present (Supplementary Table S1). Strains were clustered based on the presence/absence of these virulence determinants and they grouped largely according to their serogroup and serovar, forming separate branches in the dendogram (**Figure 1**). The *S.* Typhimurium isolates clustered into two subclades within their branch, according to presence or absence of plasmid virulence genes (**Figure 1**). Thus three broad virulence genotype groupings of *S.* Typhimurium were identified: isolates without the virulence plasmid (*n* = 36), isolates with the virulence plasmid (*n* = 8), and an isolate with both the plasmid and SGI-1 (*n* = 1). Virulence plasmid genes were also detected in group D *S. enterica* isolates.

Chromosomal and islet genes were highly conserved amongst isolates (**Figure 1**; Supplementary Table S1). Some genes were present in a specific serovar, e.g.*, lpfD* (encoding a fimbrial protein) was present in *S.* Infantis, *S.* Typhimurium, and serogroup O:9 (D1) isolates. As expected, there was a high degree of conservation of genes located on SPI-1, SPI-2, SPI-3, SPI-4, and SPI-5, and most genes were present in the majority of isolates. The *vexE* gene from *S. typhi* specific SPI-7 was absent in all isolates. The SPI-10 genes *sefA* and *sefR* were only detected in group D *Salmonella* isolates. The *cdtB* gene, encoding a cytolethal distending toxin, was present in the *S.* Montevideo isolates only.

There was also variation in the presence of prophage genes. The Gifsy-1 associated gene *gogB* was present in 100% of *S.* Typhimurium isolates and in 14 other isolates. The Gifsy-2 associated gene *sodC1* was detected in the *S.* Typhimurium and 0:9 (D1) serogroup isolates. The Gifsy-3 associated gene *sspH2* was present in *S.* Infantis, 0:9 (D) serogroup isolates, and 42/45 of *S.* Typhimurium isolates. The prophage gene *sopE1* (encoding an effector protein) was present in 19/46 *S.* Typhimurim and 13/13 serogroup O:9 (D1) isolates. The Fels-1 gene *sodCIII* was only present in LT2.

### Mortality of *Galleria mellonella* Following Infection with *S. enterica*: Dose and Isolate Dependence

**FIGURE 2:**
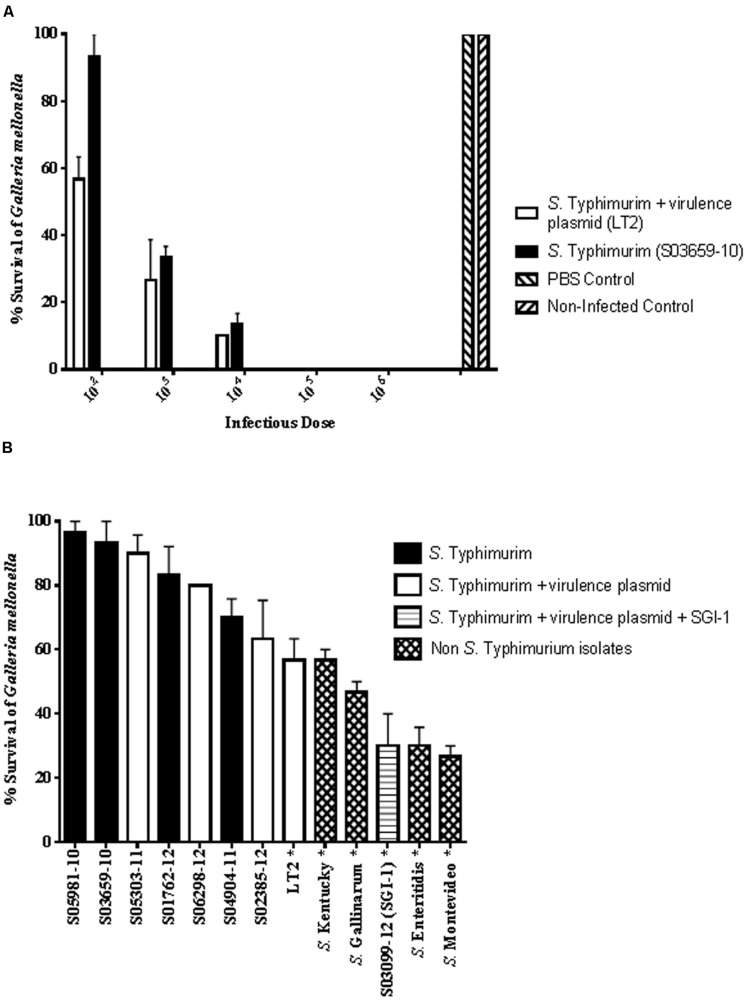
**(A)** Dose-dependent killing of *G. mellonella* after *S.* Typhimurium infection. Strain LT2 (with virulence plasmid; white bar) and isolate S03659-10 (without virulence plasmid; black bar) were inoculated with decreasing bacterial loads ranging from 10^6^ to 10^2^ CFU and larvae were scored for survival at 24 h post-infection. PBS injected and non-infected controls are also shown. Data as shown are mean results of three independent experiments and error bars represent standard deviation. **(B)** *G. mellonella* survival following infection with different *S. enterica* isolates. Isolates tested comprised four *S.* Typhimurium without the virulence plasmid (black bars); four *S.* Typhimurium with the virulence plasmid (white bars); one *S.* Typhimurium with the virulence plasmid and SGI-1 (horizontal stripes); and four non- *S.* Typhimurium isolates as indicated (hatched bars). Isolates with a significant difference (*P* < 0.05) in larval survival compared to the uninfected control are indicated with an asterisk. Data as shown are mean results of three independent experiments and error bars represent standard deviation.

To assess the potential differences in the virulence pathotype of isolates a *G. mellonella* pathogenisis model was used. Dose-dependent mortality of larvae was observed and at 10^2^ CFU/larva there was a significant difference (*P* < 0.018) in the survival of larvae infected with S03659-10 (mean survival 93.3%; range 80–100%) compared to those infected with LT2 (mean survival 56.7%; range 50–70%; **Figure 2A**). Infection with higher CFU/larva resulted in higher, up to 100%, larval mortality rates (**Figure 2A**). For both strains, enumeration of the intracellular bacteria in larvae infected with 10^2^ CFU showed large increases, with up to a seven log increase in bacterial numbers over the inoculum (Supplementary Table S2). An additional 11 isolates were subsequently tested in the *G. mellonella* pathogenesis model at an infectious dose of 10^2^ CFU/larva and scored for larval survival (**Figure 2B**). Seven of these isolates represented the three *S.* Typhimurium virulotypes identified by microarray: three with the virulence plasmid (S05303-11, S06298-12, S02385-12); three without the plasmid (S05981-10, S01762-12, S04904-11); and one with the virulence plasmid and SGI-1 (S03099-12). Also included were four non-*S.* Typhimurium isolates: one *S.* Montevideo (L00776-10); a multidrug resistant *S.* Kentucky (S02107-12); and two poultry invasive isolates (*S.* Enteritidis, S01079-10; *S.* Gallinarum, S05886-06).

The mean *G. mellonella* survival rate differed between the 13 isolates and a significant difference (*P* < 0.05) was obtained for six strains compared to the uninfected control: the four non-*S.* Typhimurium isolates and two *S.* Typhimurium isolates (LT2 and S03099-12). A mean survival rate of 30% was obtained following infection with *S.* Typhimurium isolate S03099-12% (range 10–40%) and with the *S.* Enteritidis isolate (range 20–40%). The *S.* Montevideo isolate, carrying the *cdtB* gene, resulted in the lowest survival rate for *Galleria* (mean 26.7%; range 20–30%). The mean survival rates of the remaining seven *S.* Typhimurium isolates did not differ significantly from the uninfected control, and these included three with virulence plasmid genes and four without. There were 11 virulence genes variably present in the nine *S.* Typhimurium isolates tested in the *G. mellonella* model. The effector *sopE1* was only present in 3/7 *S.* Typhimurium isolates with a low pathogenicity phenotype but there were no other virulence genes uniquely present in this group. LT2 was the only isolate harboring *sodCIII*, whereas only S03099-12 carried *hldD*_DT104 (a marker for DT104) and STM14_1441 (encoding a putative transcriptional regulator).

### Histopathology of *G. mellonella* Infected with *S. enterica*: Gut Degradation and the Formation of *Salmonella-*Containing Vacuoles

**FIGURE 3:**
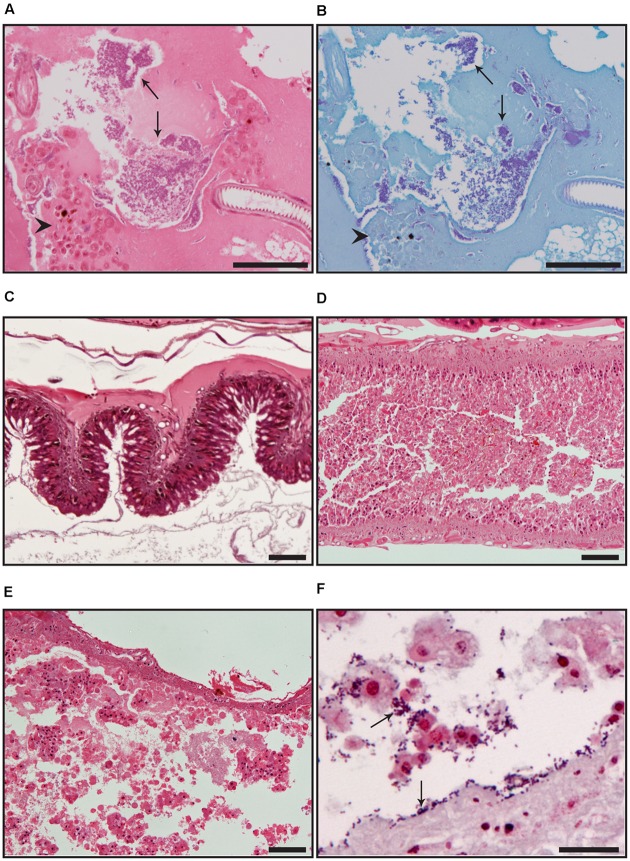
**(A,B)** Presence of bacteria in the haemolymph of *G. mellonella* following infection for 20 h with *S.* Montevideo isolate L00776-10 shown by **(A)** haematoxylin and eosin staining and **(B)** Gram stain. **(C–E)** Hematoxylin and eosin stained gut from *G. mellonella* larvae illustrating degrees of gut pathology. **(C)** Normal tissue showing intact epithelium lining the wall of the gut; larva treated with PBS 24 h post challenge. **(D)** Moderate pathological changes characterized by degenerating epithelial cells which are sloughed from the gut wall and fill the gut lumen; larva infected with *S.* Montevideo isolate L00776-10, 20 h post challenge. **(E)** Severe pathological changes characterized by complete destruction of the intestinal epithelium with necrotic cells and cellular debris present in the lumen; larva infected with *S.* Typhimurium strain LT, 24 h post challenge. **(F)** Gram stained high magnification of a gut section. Bacteria are evident in the lumen and along the gut wall which is devoid of normal epithelium; larva infected with *S.* Typhimurium isolate S03659-10, 24 h post challenge. Arrows indicate bacteria and arrowheads indicate haemocytes. An infectious dose of 10^2^ CFU/larva was used for each strain examined. Scale bar in **(A–E)** represents 100 μm; in **(F)** represents 20 μm.

The progress of *S. enterica* infection in *G. mellonella* and the ensuing histopathological changes were examined for four isolates over a 24 h period: *S.* Montevideo L00776-10 (with the lowest survival rate), *S.* Typhimurium S03659-10 (without virulence plasmid), LT2 (with virulence plasmid), and *S.* Typhimurium S03099-12 (with virulence plasmid and SGI-1). Bacteria were observed in the haemolymph by light microscopy at 5, 20, and 24 hpi and in close proximity to haemocytes in larvae infected with each isolate (**Figure 3**). Following infection there was moderate deterioration in the structure of the larval gut at 5 h for LT2, L00776-10 and S03099-12 (Supplementary Figure S1), characterized by degenerating epithelial cells which were sloughed from the gut wall and filled the gut lumen (**Figure 3**). Moderate degeneration was also noted in two larvae at 0 h post-infection with L00776-10 (Supplementary Figure S1). At 20 h severe gut degradation, characterized by complete destruction of the intestinal epithelium with necrotic cells and cellular debris present in the lumen (**Figure 3**), was noted in one larva infected with LT2, two larvae infected with L00776-10, two larvae infected with S03659-10 and all three larvae infected with S03099-12. At 24 h severe gut degradation was observed in all larvae infected with LT2, S03659-10, S03099-12, and L00776-10 (Supplementary Figure S1).

**FIGURE 4:**
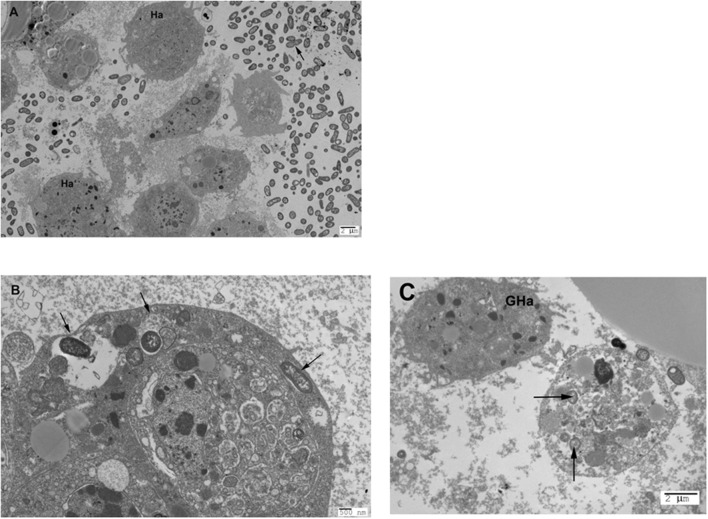
**(A)** The haemolymph of *G. mellonella* contains haemocytes and dividing bacteria 20 h post-infection. Transmission electron micrograph taken 20 h post-infection with *S.* Typhimurium isolate S03099-12; haemocytes are indicated by Ha and dividing bacteria by an arrow. **(B)** Presence of SCV-like structures in *G. mellonella* haemocytes following infection with *S.* Montevideo. Image shows a haemocyte extracted from haemolymph 20 h post-infection with *S.* Montevideo isolate L00776-10. SCV-like structures are indicated by arrows. **(C)** Granular haemocyte and haemocyte containing SCV-like structures in *G. mellonella* following infection with *S.* Typhimurium. Image shows a haemocyte containing SCV-like structures (indicated by arrows) and a granular haemocyte without SCV (indicated by GHa). Extracts were prepared from haemolymph 24 h post-infection with *S.* Typhimurium isolate S03099-12.

TEM showed the presence of haemocytes and dividing bacterial cells in the haemolymph at 20 and 24 hpi (**Figure 4A**). Furthermore, haemocytes containing one or more small discreet vacuoles in which one (or rarely two) bacteria resided were observed by TEM at 20 or 24 hpi (**Figures 4B,C**) in larvae infected with S03659-10, S03099-12, or L00776-10, but not with LT2. These intracellular bodies may be *Salmonella* containing vacuoles (SCV), but it is possible that they were phagolysosomes located within granular haemocytes, as evidence of intra-cellular bacterial cell division or replication was not observed. However, granular haemocytes observed by TEM had an appearance that was distinct from that of haemocytes containing SCV (**Figure 4C**).

## Discussion

In this study we have characterized virulence and AMR genes present in 95 UK *S. enterica* field isolates from 11 serovars. The diversity of AMR genes detected was similar to that reported previously for *S. enterica* isolates from UK livestock (Liebana et al., 2004; Randall et al., 2004; Anjum et al., 2011) and one isolate harbored SGI-1 which is commonly found in *S.* Typhimurium DT104 (Briggs and Fratamico, 1999). Carriage of large numbers of resistance genes (<9) was limited to *S.* Typhimurium mainly from pigs in which multidrug resistance is common (Anjum et al., 2011), and to the *S.* Kentucky isolate from turkey. Carriage of conserved sets of AMR genes associated with mobile genetic elements such as plasmids and integrons was noted in many isolates. For example, *strB*, *bla*_TEM_, *sul2*, and *tet*(B), which generally reside together on a chromosomal island (Hopkins et al., 2010) but have also been described on conjugable elements (Anjum et al., 2011), were detected in 25 *S.* Typhimurium isolates. The genes *aadA1*, *cmlA1*, *dfrA12*, and *sul3* often reside together in integrons located on plasmids (Garcia et al., 2014) and were present in 6 *S.* Typhimurium isolates and the *S.* Kentucky isolate. Notably, isolate *S.* Typhimurium S01762-12 carried both of these sets of AMR genes. The two *S.* Paratyphi B var. Java isolates, recovered from imported chicken meat, carried *aadA1*, *dfrA1*, and the class 2 integron, which can be present following integration into the chromosome of poultry isolates (Miko et al., 2003). This carriage of genes encoding resistance to antibiotics used in clinical or veterinary medicine predicts that isolates would be recalcitrant to certain antibiotic therapies following infection. However, it is the presence of virulence determinants that help predict the pathogenic capacity of the isolates and we examined this capacity by virulence genotyping and pathotyping.

There was considerable conservation of the virulence gene content amongst isolates with SPI −1 to −5 and many chromosomal genes conserved in all isolates, as previously reported (Anjum et al., 2005; Litrup et al., 2010; Beutlich et al., 2011; Zou et al., 2011; Suez et al., 2013; Figueiredo et al., 2015). However, differences between serovars were noted, e.g., SPI-10 genes were only detected in group D *Salmonella* isolates, in concordance with other studies (Suez et al., 2013; Figueiredo et al., 2015). Intra-serovar differences in gene carriage were most notable for *S.* Typhimurium, chiefly arising from the presence/absence of virulence plasmid genes, the variable presence of which is well documented (Litrup et al., 2010; Suez et al., 2013; Figueiredo et al., 2015). Virulence genes residing on prophages also showed some variation between isolates, as noted by others (Litrup et al., 2010; Beutlich et al., 2011; Suez et al., 2013; Figueiredo et al., 2015), indicating that these elements make a noteworthy contribution to the plasticity of the *S. enterica* genome.

To explore functional differences in virulence gene content we employed the *G. mellonella* pathogenesis model and investigated the virulence pathotype of 13 isolates, selected to represent the three virulence genotypes of *S.* Typhimurium identified by microarray, and four serovars with animal or public health significance. We demonstrated a dose-dependent effect of *S. enterica* infection on the mortality of the larvae, consistent with other studies (Bender et al., 2013; Viegas et al., 2013). Infection with each of the four non-*S.* Typhimurium isolates resulted in high mortality of the larvae. These isolates included an egg-invasive *S.* Enteritidis (Arnold et al., 2014) and a *S.* Gallinarum isolate, a serovar that is highly invasive in chickens and can also infect parasitic red mites (*Dermanyssus gallinae*), a vector for blood-borne *Salmonella* transmission in poultry houses (Zeman et al., 1982). The *S.* Montevideo isolate harbored *cdtB*, which encodes a cytolethal distending toxin that functions in a tripartite complex to cause cell cycle arrest (Mezal et al., 2014). *cdtB* was first described in *S.* Typhi but subsequently identified in a number of NTS serovars including *S.* Montevideo (Suez et al., 2013; Mezal et al., 2014; Figueiredo et al., 2015). The presence of this toxin may have contributed to *S.* Montevideo virulence in *G. mellonella*, as was previously shown for an *S.* Typhimurium isolate harboring *cdtB* (Figueiredo et al., 2015). The significant pathogenicity of the multidrug resistant *S.* Kentucky isolate is notable as it was phenotypically and genotypically similar to epidemic isolates that have been reported in humans and poultry in Europe and Africa (Le Hello et al., 2013).

Infection of *G. mellonella* with a *S.* Typhimurium isolate harboring both the virulence plasmid and SGI-1 resulted in low survival rates which were significantly different to the uninfected control. SGI-1 has been reported to confer increased *Salmonella* virulence and invasiveness in vertebrate hosts (Carlson et al., 2007) and may make a similar contribution during infection of *G. mellonella*. *S.* Typhimurium LT2, a well characterized strain harboring several virulence determinants including the virulence plasmid and the Fels-1 prophage (McClelland et al., 2001), also produced significant mortality. This contrasts with the virulence phenotype of LT2 in experimentally infected mice, in which it is attenuated for systemic infection, e.g., in the liver and spleen (Swords et al., 1997; Wilmes-Riesenberg et al., 1997). Importantly, however, LT2 demonstrates several other virulence phenotypes, including the ability to adhere to and invade human epithelial (Wilmes-Riesenberg et al., 1997; Jolivet-Gougeon et al., 2003) and colon cells (Roberts et al., 2013; Figueiredo et al., 2015) *in vitro*, as well as survive and replicate within macrophages (Wilmes-Riesenberg et al., 1997). Additionally, *Salmonella* strains SL1344 and ATCC 14028, which are virulent in mice, also demonstrate virulence in *Galleria* (Bender et al., 2013; Viegas et al., 2013). Interestingly, infection with the remaining seven *S.* Typhimurium isolates, including three possessing the virulence plasmid, did not significantly increase larval mortality. Thus, although the virulence plasmid encodes functions that enable survival in macrophages and facilitate systemic infection (Gulig et al., 1998; Haneda et al., 2012), it may not function in insect haemocytes in the same manner and/or other virulence determinants (e.g., SGI-1, Fels-1, or others) may be required for extensive *S.* Typhimurium pathogenesis in this model.

Histopathological examination of infected larvae demonstrated that *S. enterica* replicates successfully in the haemolymph, correlating with the enumeration data that showed large increases in bacterial numbers 24 hpi. In vertebrates the formation of SCV in macrophages is an important stage for the onset of systemic infection. We have demonstrated the presence of SCV-like structures in *G. mellonella* following infection with *S. enterica*. The presence of SCVs indicates that the *Salmonella* virulence factors which enable adhesion and invasion, such as those encoded on SPI-1, function in *G. mellonella*; a hypothesis supported by previous work in which a *S.* Infantis isolate lacking 38 SPI-1 genes gave reduced mortality in this model (Figueiredo et al., 2015). We did not observe SCV following infection with LT2, but given the significant pathogenicity this strain demonstrated in the larvae it may have been due to insufficient sampling for TEM with this sample. The absence of evidence for *S. enterica* replication within the haemocytes may also have arisen from low sampling numbers. Alternatively, the *Salmonella* may have been unable to replicate as they were trapped in phagolysosomes located within granular haemocytes, although this is less probable as the haemocytes containing SCV-like structures were distinctly different to the granular haemocytes observed by TEM. It is also possible that virulence factors that promote intracellular replication and survival, such as those located on the virulence plasmid and SPI-2, do not function in haemocytes as they do in macrophages and warrants further investigation. However, haemocytes are able to support the replication in vacuoles of other bacteria, such as *L. pneumophila* (Harding et al., 2012).

## Conclusion

We have used virulence and AMR genotyping to provide an assessment of the pathogenic potential of a variety of *S. enterica* serovars and isolates and their susceptibility to antibiotic therapy. Although there was no correlation between AMR carriage and virulence determinants present, it is pertinent to determine AMR gene carriage as it will impact therapeutic options. Thus the microarray continues to provide a rapid and easy-to-use epidemiological surveillance tool that could be used to help inform intervention measures and can be more accessible for many diagnostic laboratories than whole genome sequencing. We have shown that *Salmonella* isolates of clinical importance in humans and poultry can replicate and produce significant pathology in *G. mellonella*, indicating that this virulence model may replicate some aspects of *Salmonella* infection in mammals. However, as noted by Kavanagh and Reeves (2004) this model may not be suitable for the study of disease processes or microbial dissemination pathways that are specific to mammals. For example, the insect haemolymph fills the body cavity and may not provide the same barrier to systemic infection as the closed circulatory system of mammals. Furthermore confirmatory work, including a direct comparison of LT2 and a virulent strain such as SL1344 at the same infectious dose, is required before the *Galleria* model can be used to help predict the virulence potential of *Salmonella* for public or animal health. To our knowledge this study reports the first demonstration of SCV-like structures in *G. mellonella* and supports the further investigation of the potential of this model for preliminary investigation of the pathogenicity of *S. enterica*, due to the low cost and simplicity of the procedure.

## Author Contributions

Conceived and designed the experiments: MA, RD, and RC. Performed the experiments: RC, KV, MB, JS, WC, and TS. Analyzed the data: RC, KV, and MA. Wrote the paper: RC, RD, and MA.

## Conflict of Interest Statement

The authors declare that the research was conducted in the absence of any commercial or financial relationships that could be construed as a potential conflict of interest.
