# Supplementary material for: Virulence Characterisation of Salmonella enterica Isolates of Differing Antimicrobial Resistance Recovered from UK Livestock and Imported Meat Samples
Source: Front Microbiol. 2016 May 2;7:640. doi: 10.3389/fmicb.2016.00640 (PMC4852480; doi:10.3389/fmicb.2016.00640)
Supplement: Supplementary file 2 [file Table_2.DOCX]

Data Sheet S1. Enumeration of the intracellular bacteria in larvae infected with an inoculation dose of 1x10^2^ CFU / larva of isolate LT2 or S03659-10.

Experiment 1

| **Strain** | **Galleria alive/dead** | **Recovered colony count** | **Log increase** |
| --- | --- | --- | --- |
| LT2 (larva 1) | Alive | 1.62 x 10^9^ CFU | 7 |
| LT2 (larva 2) | Alive | 1.07 x 10^9^ CFU | 7 |
| LT2 (larva 3) | Dead | 4.33 x 10^8^ CFU | 6 |
| S03659-10 (larva 1) | Alive | 7.50 x 10^5^ CFU | 3 |
| S03659-10 (larva 2) | Alive | 5.50 x 10^7^ CFU | 5 |
| S03659-10 (larva 3) | Dead | 4.33 x 10^8^ CFU | 6 |

Experiment 2

| **Strain** | **Galleria alive/dead** | **Recovered colony count** | **Log increase** |
| --- | --- | --- | --- |
| LT2 (larva 1) | Alive | 2.67 x 10^4^ CFU | 2 |
| LT2 (larva 2) | Alive | 1.32 x 10^5^ CFU | 3 |
| LT2 (larva 3) | Dead | 1.82 x 10^8^ CFU | 6 |
| S03659-10 (larva 1) | Alive | 1.42 x 10^8^ CFU | 6 |
| S03659-10 (larva 2) | Alive | 5.00 x 10^7^ CFU | 5 |
| S03659-10 (larva 3) | Dead | 6.00 x 10^8^ CFU | 6 |

Experiment 3

| **Strain** | **Galleria alive/dead** | **Recovered colony count** | **Log increase** |
| --- | --- | --- | --- |
| LT2 (larva 1) | Dead | 3.5 x 10^8^ CFU | 6 |
| LT2 (larva 2) | Dead | 6.0 x 10^7^ CFU | 5 |
| LT2 (larva 3) | Dead | 4.3 x 10^8^ CFU | 6 |
| S03659-10 (larva 1) | Alive | 7.2 x 10^4^ CFU | 2 |
| S03659-10 (larva 2) | Alive | 6.7 x 10^4^ CFU | 2 |
| S03659-10 (larva 3) | Dead | 5.8 x 10^8^ CFU | 6 |
