# Supplementary material for: Virulence Characterisation of Salmonella enterica Isolates of Differing Antimicrobial Resistance Recovered from UK Livestock and Imported Meat Samples
Source: Front Microbiol. 2016 May 2;7:640. doi: 10.3389/fmicb.2016.00640 (PMC4852480; doi:10.3389/fmicb.2016.00640)

Figure S1. Aggregate gut degradation score of three larvae at 0, 5, 20 and 24 hours post-infection with isolate LT2, S03099-12, S03659-10 or L00776-10. For each isolate the extent of degeneration of the gut structure in the larvae was scored as 0 (no change/normal), 1 (moderate) or 2 (severe) and plotted as the total score obtained from the three larvae at each time-point.

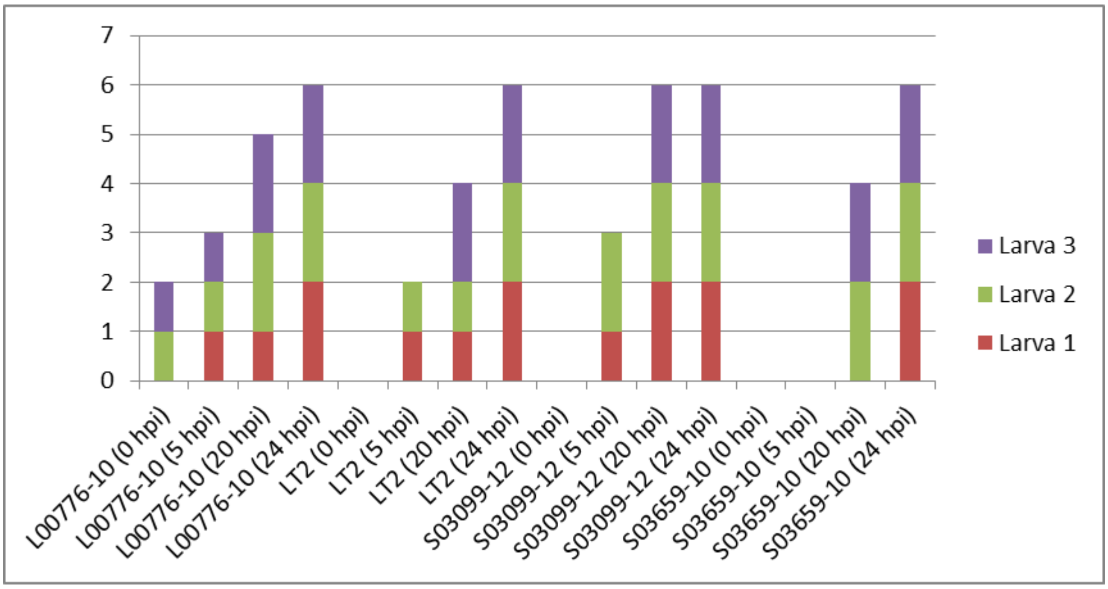

Supplement: Supplementary file 3 [file Image_1.PDF]
